# Training profile of intensive care nurses in Brazil: cross-sectional study

**DOI:** 10.1590/0034-7167-2023-0460

**Published:** 2024-12-16

**Authors:** Thais Oliveira Gomes, Fernanda Berchelli Girão, Tágora do Lago Santos, Matheus Henrique Silva, Erika Azevedo Portes, Clayton Lima Melo, Marcus Vinicius Melo de Andrade

**Affiliations:** I Universidade Federal de Minas Gerais. Belo Horizonte, Minas Gerais, Brazil; II Universidade Federal de São Carlos. São Carlos, São Paulo, Brazil; III Universidade Federal do Piauí, Hospital Universitário. Teresina, Piauí, Brazil; IV Faculdade de Saúde Santa Casa BH. Belo Horizonte, Minas Gerais, Brazil; V Pontifícia Universidade Católica de Minas Gerais. Belo Horizonte, Minas Gerais, Brazil

**Keywords:** Intensive Care Units, Professional Practice, Education, Nursing, Education, Nursing, Graduate, Critical Care Nursing., Unidades de Cuidados Intensivos, Práctica Profesional, Educación en Enfermaría, Educación de Postgrado en Enfermería, Enfermaría de Cuidados Críticos.

## Abstract

**Objectives:**

to describe the training profile of Brazilian intensive care nurses.

**Methods:**

a cross-sectional study carried out in two stages: a structured, self-administered questionnaire; mapping of the national supply of *lato sensu* postgraduate courses. Data was collected on the sociodemographic profile, training process and characterization of the courses.

**Results:**

in the first stage, 202 respondents were obtained. The majority were women (79.2%), aged between 26 and 45 (80.7%), graduated less than 5 years ago (44%), through *lato sensu* postgraduate courses (55.5%), which were marked by the absence of laboratory practice (57.5%) and guided tours (42.5%). In the second stage, 457 courses were identified, with face-to-face teaching (58.9%), a workload of 360 to 420 hours (51.2%), a duration of up to 6 months (41.8%) and variation in the sub-area of training.

**Conclusions:**

there was a predominance of professionals graduating from *lato sensu* post-graduate courses, with essentially theoretical teaching and heterogeneity in terms of modality, workload and sub-area of training.

## RESUMEN

**Objetivos:**

describir el perfil de formación de los enfermeros de terapia intensiva brasileños.

**Métodos:**

estudio transversal realizado en dos etapas: cuestionario estructurado y autoadministrado; mapeo de la oferta nacional de cursos de postgrado *lato sensu*. Se recogieron datos sobre el perfil sociodemográfico, el proceso de formación y la caracterización de los cursos.

**Resultados:**

en la primera etapa se obtuvieron 202 encuestados. La mayoría eran mujeres (79,2%), con edades comprendidas entre 26 y 45 años (80,7%), con menos de 5 años de formación (44%), a través de un curso de postgrado *lato sensu* (55,5%), que se caracterizó por la falta de prácticas de laboratorio (57,5%) y visitas guiadas (42,5%). En la segunda etapa, se identificaron 457 cursos, con enseñanza presencial (58,9%), carga horaria de 360 a 420 horas (51,2%), duración de hasta 6 meses (41,8%) y variación en la subárea de formación.

**Conclusiones:**

hubo predominio de profesionales egresados de cursos de postgrado *lato sensu*, con enseñanza esencialmente teórica y heterogeneidad en cuanto a modalidad, carga horaria y subárea de formación.

## INTRODUCTION

Serious illnesses or health conditions that are potentially unstable and life-threatening require continuous monitoring and follow-up in a specific sector with a specialized care team. These are the so-called critically ill patients who are admitted to intensive care units and require constant monitoring and complex care, demanding a highly qualified nursing team^(1)^. Intensive Care Units (ICUs) are areas for the hospitalization of these patients, requiring specialized professionals on a continuous basis, as well as specific materials and technologies^(2)^.

Since 2020, the COVID-19 pandemic has highlighted the shortage of nurses qualified to work in intensive care, as well as the difficulty of identifying professionals qualified to care for critically ill patients in scenarios where demand exceeds supply, such as in disaster situations^(3)^.

The literature shows that the continuing education of intensive care nurses, as well as their professional qualifications, are determinants of favorable clinical outcomes for ICU patients, such as a reduction in length of stay, time to treatment, costs, mortality and increased patient satisfaction^(4)^. However, not all nurses are qualified to work in the intensive care setting and the skills and abilities required for this type of nursing care are irreplaceable^(5)^.

In Brazil, intensive care nurses are trained in three ways: (1) training through *lato sensu* postgraduate courses, which consist of specialization programs offered by Higher Education Institutions, which have a minimum duration of 360 hours, with a certificate at the end of the course; (2) multiprofessional residency in intensive care nursing, as established by the National Commission for Multiprofessional Residency in Health; (3) a professional certification process for intensive care nurses by obtaining the title of intensive care nurse specialist awarded by the Brazilian Association of Intensive Care Nursing (ABENTI), in the adult, pediatric and neonatal modalities, after passing the theoretical and practical title test^(6)^.

The Ministry of Health, through Collegiate Board Resolution (RDC) n 26/2012^(7)^, which amends items III and IV of article 14 of RDC n 07/2010^(2)^, establishes the ratio of one care nurse for every ten beds or fraction thereof, and does not require a minimum level of qualification to work in intensive care, with proof of qualification being mandatory only for nursing coordination, as set out in RDC 137/2017^(8)^.

In addition, the Federal Nursing Council (Cofen) regulates through Cofen Resolution n 625/2020^(9)^ the procedures for registering *lato sensu* and stricto sensu postgraduate qualifications, as well as those provided by societies, associations or colleges of nursing specialists, and approves the list of specialties. The first article of this resolution states that nurses must register their qualifications, which is not usually required by hospitals when hiring intensive care nurses, so there is no legal requirement for proof of specialty for professional practice in intensive care.

Still with regard to professional training, in addition to the number of professionals who are specialists, it is necessary to look at the quality and product of this training, i.e. whether the nurse who holds the title of specialist in intensive care is in fact capable of providing complex, quality care. A European study has shown great variation in intensive care nursing postgraduate specialization programs in terms of duration, variations in eligibility, assessment requirements and lack of access to educational resources, which can impact on the quality of teaching and significantly influence the quality of care provided by graduates of these programs^(10)^.

Other authors have pointed out that the variation in the care provided by intensive care nurses and the lack of homogeneity and systematization may be determining factors for the differences observed in the results of patients admitted to intensive care with similar disease profiles^(11)^.

Thus, considering that the care provided by a nurse specialized in intensive care has an impact on patient safety and favorable clinical outcomes, the different types of training of intensive care nurses in Brazil, as well as the fact that specialization is not required for nurses to work in this setting, there is a need to know the training profile of intensive care nurses in Brazil, understanding the paths taken by intensive care nurses throughout their professional training, as well as information on who these professionals are in terms of sociodemographic aspects, time and characteristics of training, as well as details of intensive care nursing training courses in Brazil.

## OBJECTIVES

To describe the training profile of intensive care nurses in Brazil.

## METHODS

### Ethical aspects

The study was conducted in accordance with national and international ethical guidelines and approved by the Research Ethics Committee of the Federal University of Minas Gerais, whose opinion is attached to this submission. Informed consent was obtained from all the individuals involved in the study online.

### Study design and period

This is a quantitative descriptive cross-sectional study on the educational profile of intensive care nurses in Brazil. The research data was collected from January to July 2023. The manuscript was based on the STrengthening the Reporting of OBservational studies in Epidemiology (STROBE) framework^(12)^.

### Population, sample and selection criteria

This study was carried out in two independent stages, the first of which was targeted at professionals who had graduated from a specialization course in intensive care nursing in the form of a multiprofessional health residency or a *lato sensu* postgraduate course; those who had been awarded the title of specialist nurse in intensive care by a specialist association registered with the Federal Nursing Council (Cofen); and intensivist nurses who were coordinators of *lato sensu* postgraduate courses in intensive care nursing and multiprofessional health residencies in intensive care nursing. In order to obtain a larger number of respondents to map aspects related to training, all intensive care nurses who met the above criteria could respond to the survey, as long as they worked in intensive care in the area of care, research, teaching or management. As this was a non-probabilistic sample, no sample calculation was made.

In the mapping stage of the *lato sensu* postgraduate courses in intensive care nursing registered with the MEC, the inclusion criteria were all courses with active registration and which had all the mandatory information on the website filled out in full.

### Study protocol

In the first stage, a structured, self-administered questionnaire was made available through an electronic form. The second stage involved mapping the national supply of *lato sensu* postgraduate courses in intensive care nursing registered with the Ministry of Education (MEC).

In the first stage, respondents could only proceed with filling in the self-administered form if they agreed to the Informed Consent Form (ICF). Both the ICF and the answers provided were made available at the end of the questionnaire, and completion was limited to one answer per participant. The link to the questionnaire, created in Google Forms, was disseminated through the mailing lists and social media of national and regional intensive care specialist societies and associations.

The questionnaire consisted of 47 closed questions and was divided into 6 sections, namely: section 1: sociodemographic data; section 2: *lato sensu* postgraduate course - coordinator; section 3: multiprofessional residency in health - coordinator; section 4: *lato sensu* postgraduate course - graduate; section 5: multiprofessional residency in health - graduate; section 6: title of nurse specialist in intensive care.

The questions in sections 2 to 5 covered the following topics: length of training, type of course taught, selection process for entry to the course, prerequisites for entry to the course, workload, provision of laboratory practice, guided tours or practical experience in a hospital institution, assessment methodology for obtaining the degree and sub-area of training. The content of the questions in section 6 covered: time taken to obtain professional certification, sub-area of the degree, methodology for obtaining the degree.

*Lato sensu* postgraduate courses are considered to be specialization programmes with a minimum duration of 360 hours, according to Art. 44, item III, of Law 9.394, of 20 December 1996^(13)^. Although it is understood that the multiprofessional health residency is a *lato sensu* postgraduate teaching modality, according to Art. 13 of Law 11.129, of June 30, 2005^(14)^, it was decided to describe them separately, considering the differences in workload, dedication regime and other aspects of training, which are the subject of this study.

For the purposes of standardization, the title is understood as the process of professional certification of the title of nurse specialist in intensive care granted by an association of specialists registered with COFEN.

Professionals with more than one degree in intensive care should enter this information at the beginning of the form and then be directed to the answers corresponding to each of their degrees. The coordinators of *lato sensu* postgraduate courses and multiprofessional residencies, even if they were graduates, were encouraged to provide information about the course they were coordinating in the sections intended for this purpose, also answering sociodemographic questions and the time and type of their training.

In the second stage of the study, the e-MEC website was consulted, in the Advanced Consultation tab, selecting the search for Specialization Course and using the filters “intensive care nursing” and “ICU nursing”. This is publicly accessible data from the official database of courses and Higher Education Institutions (HEIs), whose information is entered by the HEIs themselves, and they are responsible for the veracity of the data provided. When consulting e-MEC, the following information was extracted: the course’s teaching modality, the region where it is offered, the workload, the sub-area (adult, neonatal, pediatric or joint training with another specialty) and the duration of the course.

### Analysis of results and statistics

The data from the self-administered form and from the e-MEC consultation were gathered in a Microsoft Excel spreadsheet version 16.7, analyzed using descriptive statistics and presented in tables in absolute numbers and percentages.

For the analysis of sections 2 to 5 of the self-administered questionnaire, the responses of the postgraduate and residency coordinators were analyzed together with those of the graduates, as they were the same questions.

## RESULTS

A total of 207 participants accessed the questionnaire and of these, 202 were part of the final sample, agreeing to the ICF and answering the questionnaire in full.

Of the 202 intensivist nurses who responded to the questionnaires, 189 (93.5%) were graduates of *lato sensu* specialization courses, multiprofessional health residency or professional degrees and only 8 (4%) were coordinators of *lato sensu* postgraduate courses and 5 (2.5%) coordinators of multiprofessional health residency programs.

Table 1 shows the characterization of the respondents in terms of sociodemographic profile, type and length of training in intensive care. It is possible to see that the majority of the intensive care nurses taking part in the research are women (79.2%), aged between 26 and 45 (80.7%), graduated from a *lato sensu* postgraduate course (55.5%) and less than 5 years ago (44%). With regard to the region of the country, there was a predominance of training in the southeast and northeast (67.8%).

**Table 1:** Sociodemographic profile, type and length of training in intensive care of the respondents in stage 1 of this study (N=202), Belo Horizonte, Minas Gerais, Brazil, 2023

Category	n	(%)
Sex Female Male	160 42	79.2 20.8
Age 18 - 25 years 26 - 35 years 36 - 45 years 46 - 55 years > 55 years	10 64 99 25 4	5 31.7 49 12.3 2
Region of the country in which were graduated South Southeast North Northeast Midwest	23 73 24 64 18	11.4 36.1 11.9 31.7 8.9
Type of intensive care training *Lato sensu* postgraduate courses Multiprofessional health residency Title More than one training course^*^	112 30 38 22	55.5 14.8 18.8 10.9
Time spent training in intensive care Less than 5 years ago 5 - 10 years Over 11 years old	100 67 60	44 29.5 26.5

* *More than one qualification: lato sensu postgraduate degree and title or lato sensu postgraduate degree and multiprofessional residency or multiprofessional residency and title or all three modalities.*

Below are details related to each of the types of training in intensive care (post-graduate *lato sensu*, multiprofessional health residency and degree) reported by the respondents in stage 1 of this study. Considering the possibility of reporting more than one type of training in intensive care, 227 responses were obtained from the total of 202 respondents, 127 of which referred to *lato sensu* postgraduate courses, 40 to multiprofessional residencies and 60 to degrees.

With regard to training through *lato sensu* postgraduate courses, Table 2 shows that the courses were marked by face-to-face teaching (78.7%), with workloads ranging from 360 to 420 hours (76.4%) and a lack of laboratory practice (57.5%) and supervision (42.5%), presenting an essentially theoretical character, which is also perceived through the assessment methodology for obtaining the title. In this case, the respondent was allowed to mark more than one item, and the following were cited: presentation of the Course Conclusion Paper (62%), submission of a scientific article (11%), minimum attendance at classes (22.5%) and others such as tests, passing all the courses offered and an average grade (4.5%). Although there was a predominance of courses aimed at specific training in one sub-area - adult or neonatal or pediatric - which was present in 70.1% of the responses, it is noticeable that there are courses with training in more than one sub-area, with joint training in adult, neonatal and pediatric intensive care (13.4%) and training associated with areas related to intensive care, such as cardiology, urgency and emergency and trauma (16.5%) being cited.

**Table 2:** Characterization of *lato sensu* postgraduate nursing courses in intensive care identified in stage 1 of the study (n=127), Belo Horizonte, Minas Gerais, Brazil, 2023

Description	n	(%)
Teaching method Face-to-face Distance learning Hybrid	100 14 13	78.7 11 10.3
Selection process to enrol in the course Yes No	83 44	65.4 34.6
Course workload Up to 360 hours 360 - 420 hours > 420 hours	40 57 30	31.6 44.8 23.6
Laboratory practice Yes No	54 73	42.5 57.5
Practical experience or supervision Yes (elective) Yes (mandatory) Yes (visits in specific subjects) No	5 34 34 54	4.1 26.7 26.7 42.5
Specific training in a sub-area Yes No (adult, neonatal and pediatric) No (intensive care and related areas)	89 17 21	70.1 13.4 16.5
Assessment methodology for the title Presentation of final paper Submission of a scientific article in an indexed journal Minimum class attendance Other^*^	108 19 39 8	62 11 22.5 4.5

* *Other: exams, pass in all subjects offered, average grade.*

With regard to training through a multi-professional health residency, of the 40 responses obtained, 100% of the intensive care nurses took part in a selection process to enter the course, with CV analysis, an interview and a multiple-choice and/or essay test being reported. Twenty-five (62.5%) of the nurses reported that no laboratory practice was offered. As for the specialty subarea, the predominant training was in the adult intensive care subarea (25) (62.5%), followed by mixed training in adult, neonatal and pediatric intensive care (14%), specific training in neonatal and pediatric (9%), as well as intensive care and related areas (14%), in the same way as the *lato sensu* postgraduate course.

As for training by degree, of the 60 responses obtained, there was a predominance of training in the adult sub-area - 50 (83.3%), followed by pediatrics - 6 (10%) and neonatal - 4 (6.7%).

The data relating to stage 2 of this study is presented below. A total of 457 *lato sensu* postgraduate courses in intensive care nursing with active status in e-MEC were identified. The majority of teaching methods were face-to-face (58.9%), with a predominance of courses in the southeast (48.4%), a workload of 360 to 420 hours (51.2%) and a predominant duration of up to 6 months (41.8%). When informed, there was a wide variation in the description of the sub-area of the course, with courses being identified exclusively for adult training (9.8%), neonatology (3.3%), as well as neonatology and pediatrics concurrently (20.8%), also in adult, neonatology and pediatrics (3.1%) and associated with other areas of critical patient care, such as urgency and emergency, trauma, cardiology and neurology (14.2%).

The results of the second stage of the study are shown in Table 3.

**Table 3:** Data from *lato sensu* postgraduate courses in intensive care nursing with active status in e-MEC identified in stage 2 of the study (N=457), Belo Horizonte, Minas Gerais, Brazil, 2023

Course description	n	(%)
Type of teaching Face-to-face Distance	269 188	58.9 41.1
Region of supply South Southeast North Northeast Midwest More than one region	65 221 31 63 33 44	14.2 48.4 6.8 13.8 7.2 9.6
Working hours Up to 360 hours 360 - 420 hours > 420 hours > 5000 hours	130 104 221 2	28.4 22.8 48.4 0.4
Duration > 6 meses 6 - 12 months 13 - 18 months 19 - 24 months	191 105 117 43	41.8 23 25.6 9.4
Course sub-area Not informed Adult Neonatal Pediatric Neonatal and pediatric Adult, neonatal and pediatric Adult and pediatric Adult and neonatal Intensive care and other subspecialties (urgency and emergency, trauma, cardiology, neurology)	216 45 15 0 95 14 1 6 65	47.3 9.8 3.3 0 20.8 3.1 0.2 1.3 14.2

## DISCUSSION

With regard to the sociodemographic profile, the data from this study revealed that the majority of participants were women aged between 36 and 45. These results are similar to those reported by a multicenter study on the profile of intensive care nursing professionals carried out in South America, with the participation of Argentina, Colombia, Peru and Brazil, where the female prevalence in the four countries was 83% and in Brazil it was 79%, while the global median age was 36 years and the Brazilian median age was 29 years^(15)^. In more distant countries such as Japan, the predominance of women (77.7%) and young people aged between 20 and 29 is also found, although there are different economic and cultural aspects^(16)^.

The data from this study shows that the majority of Brazilian intensive care nurses are young in their specialty, with up to five years of training in intensive care. Time spent training in intensive care is essential for intensive care nurses to develop reasoning skills and clinical judgment, strengthening their practice and promoting greater safety in their professional work^(15)^. Regarding the length of time working in intensive care and the retention of nurses working in the specialty, an American study discusses the difficulty in retaining nurses in intensive care, which may be associated with factors such as burnout and work overload, circumstances aggravated by the pressure imposed during the COVID-19 pandemic on these units^(5)^. Some authors problematize that there is a tendency for intensive care nurses to transition to positions outside intensive care units as they advance in their training^(17)^. Thus, strategies are suggested for retaining intensive care nurses working in the specialty, in the different areas of activity (management, teaching/research and/or care), such as: recognition, respect and appreciation of the important role and high levels of training required to work in this setting; active participation in the unit’s activities, with involvement in complex decision-making such as in end-of-life situations, pain and comfort management, guided protocols, multidisciplinary rounds; encouraging intellectual and professional development; encouraging learning opportunities and involvement in training activities such as residency; continuous leadership training with feedback instructions, seeking to promote mutual growth in the team, among others^(5)^.

In this regard, it is important to mention that, although fewer in number, a reasonable number of nurses with more than one type of training in intensive care were identified (*lato sensu* post-graduation and degree or *lato sensu* post-graduation and multiprofessional residency or multiprofessional residency and degree or all three). This finding may be related to the fact that nurses involved in teaching, research and intensive care management, as well as those involved in care activities, were allowed to express their opinion. A cross-sectional study evaluating the competencies of nurses working in intensive care in tertiary hospitals in Japan found that professionals with a greater number of degrees in intensive care scored better in competencies related to decision-making, collaboration and nursing interventions^(16)^. Therefore, maintaining this professional in activities related to the care of critically ill patients, whether in bedside assistance, management positions or teaching and research positions focused on the area, is essential to ensure that the complex care and advanced knowledge required to work in intensive care are guaranteed^(17)^.

As far as the training profile is concerned, considering the three types of training for intensive care nursing in Brazil, there is a predominance of *lato sensu* postgraduate training. It can also be seen that most of the courses do not have any laboratory practice hours, showing that the training is essentially theoretical, which can also be seen in the assessment methodology used to obtain the degree. Practical experience and/or guided visits to health services is mainly applicable to the multiprofessional health residency model, due to its very nature as in-service training. However, in this study this type of training was substantially lower than the others.

Still with regard to the training profile, although the face-to-face teaching modality was identified in both stages of the study as the majority of postgraduate *lato sensu* courses on offer, the high percentage of distance learning courses identified in stage 2 is noteworthy. These courses are of short duration, generally up to 6 months, with workloads ranging from 360 to 420 hours.

The large number of distance learning postgraduate courses, together with the lack of courses with practical experience or guided tours, are worrying factors in terms of the quality of teaching and the development of intensive care nurses’ skills. Acquiring experience is an important part of developing intuition and professional competence, as it allows nurses to anticipate the evolution of a clinical condition and base decisions on previous experience with similar situations^(18)^. Safety in managing situations depends on the experience of the nurse. Trainers and educators in intensive care need to consider designing practical programs for inexperienced nurses that include intentional exposure to various life-threatening clinical situations inherent in intensive care nursing^(18)^. What’s more, it’s not possible to improve skills such as teamwork, handling demographic and cultural influences, interpersonal relationships with ICU patients and their families, or technology management through distance learning and without on-the-job training^(18)^. Thus, it must be considered that, since *lato sensu* postgraduate courses are the predominant type of training and most of them do not offer practical activities, there is a possibility that this type of training is insufficient in terms of acquiring the skills needed to work in an intensive care unit.

In the results found here, there is heterogeneity in the training of intensive care nurses in Brazil, from the form of access to the specialization opportunity, which can select professionals for training by means of tests, to courses that only require prior graduation as a requirement, including variations in duration, workload, teaching modality and training sub-area. Similarly, a European study identified the lack of a national education and training standard as a problem in most countries^(19)^. Difficulties range from eligibility requirements and course lengths ranging from 240 hours to 24 months, with no consistency in how students were assessed and qualified to be awarded the training, followed by a lack of protection for the title, limiting student working hours that impact teaching and training in the ICU, and a lack of access to education resources^(19)^. Also with regard to access to specialization, an Australian study found that most courses did not require previous clinical experience as a prerequisite for entry into training, which is similar to the Brazilian reality found in this study, with the only exception being the title test^(20)^.

With regard to training in more than one sub-area, an important finding of this study was the heterogeneity of the sub-area of intensive care training, ranging from courses with joint training in adults, neonatology and pediatrics, to specialization courses in intensive care and related areas, such as cardiology, neurology, urgency, emergency and trauma. Australian authors have revealed that in Australia there are various interpretations of what comprises an intensive care qualification, with some postgraduate courses offering a broad range of content to cover the areas of intensive care, cardiac care or, for some, a combination of emergency nursing, high dependency nursing and/or trauma nursing^(20)^.

In this respect, it is worth questioning the generalization of a specialization, after all, its aim should be to refine knowledge and acquire specific skills for working in a given context. However, what can be seen is a movement to include multiple clinics in a single scope of training, which points to the risk that, by trying to encompass many disciplines, the training offered will be increasingly generalist and less capable of actually promoting specialization. Still in this area, another finding that deserves reflection is the workload of less than 420 hours in most *lato sensu* postgraduate courses. Thus, although the MEC defines a minimum workload of 360 hours, the question arises as to what would be a sufficient workload to cover more than one area of knowledge and its specificity.

The Federal Nursing Council, in article 10 of Resolution No. 581/2018^(21)^ and its amendments (COFEN No. 625/2020; COFEN Nos. 065/2021 and 120/2021) point out that it is compulsory to register *lato sensu* and stricto sensu postgraduate degrees (the latter in the professionalizing modality) with the Regional Council of your jurisdiction. However, neither this registration with the Council nor the specialist title are required by health institutions to employ nurses who will work in intensive care units, with the exception of the nursing coordinator.

The annex to the legislation mentioned above, when defining the areas and sub-areas of knowledge for registering titles with the Regional Nursing Council, limits the registration of the title to the areas listed in the instrument. Thus, although there are multi-area courses, as identified in the survey, nurses can only register in the areas and sub-areas listed in the annex to Resolution No. 581/2018^(21)^. The sub-areas allowed in the registration of the Intensive Care title, item 43 of the annex of the aforementioned Resolution are only a) Adult, b) Cardiology, c) Neurology, d) Pediatrics, e) Neonatology, and sub-areas cannot be accumulated with a single specialization course.

One finding of this study that deserves attention is the predominance of training in the southeast and northeast regions identified in stage 1, as well as the significant number of courses offered in these locations seen in stage 2. This may be associated with the availability of intensive care beds in these regions, which could lead to a greater demand for specific training in this type of care. Data from the Census of the Brazilian Intensive Care Medicine Association (AMIB) on the distribution of intensive care beds in Brazil in January 2023 revealed a total of 22,618 beds in the southeast and 9,429 in the northeast, ranking first and second, respectively, in the absolute number of public and private intensive care beds in the country^(22)^.

### Study limitations

The limitation of this study is the small number of respondents to stage 1, in which data was obtained on the training of 202 intensive care nurses. However, according to official data obtained through contact with Abenti’s formal communication channels in June/2023, the total number of nurses who have been certified and obtained the title of nurse specialist in intensive care in the adult, pediatric and neonatal modalities is 405 professionals. Cofen’s data on the number of nurses with the title of specialist in intensive care in the adult, neonatal and pediatric modalities on May 31, 2023 was 11,605 professionals, according to information obtained through a request registered in Cofen’s Ombudsman system. However, this is the overall number of registered nurses without a time filter, and may therefore include professionals who are not active, such as retired professionals.

### Contributions to the field of Nursing

To our knowledge, this is the first study to present data on the number of intensive care nurses and the training profile of this specialist in Brazil.

Another relevant aspect of this study is that the data on training through *lato sensu* postgraduate courses identified in stage 1, regarding workload, teaching modality, region of offer and sub-area, are similar to those found in stage 2, when all *lato sensu* postgraduate courses with active registration with e-MEC were mapped. Thus, it can be said that there is solidity and coherence in the data obtained in the two stages of the work, with the information from stage 1 being reaffirmed in stage 2, which makes sense because, since *lato sensu* postgraduate courses are the predominant type of training, their characterization using official MEC data clarified the findings from stage 1.

Thus, this study not only opens up avenues for new studies, but also raises other questions adjacent to the topic and which explain, among other things, how training shapes the technical competence that is actually required for nurses to work in intensive care.

## CONCLUSIONS

This study described the training profile of intensive care nurses in Brazil in terms of the predominant type of training in the country, as well as sociodemographic aspects and the length of time these professionals have been trained. Details were also presented regarding each type of intensive care training in terms of workload, duration, teaching modality, entry to the course, assessment methodology and training sub-area.

The predominance of professionals training in *lato sensu* postgraduate courses found in this study, which offer essentially theoretical teaching, as well as the heterogeneity of these in terms of teaching modality, duration, workload and training sub-area demonstrate the need to define the professional competencies of intensive care nurses in Brazil.

It is therefore imperative to promote progress towards competency-based professional training, which provides an ongoing process of evaluation of intensive care nurses based on the knowledge, skills, attitudes and values expected of them in clinical practice. Furthermore, it is understood that there is a need to describe the professional competences of intensive care nurses, with a view to contributing to: a better definition of their role and attributions in their day-to-day work; a definition of the minimum essential requirements for their professional work in intensive care; the standardization of a common language for their training; an understanding of the gaps that exist in the training of this professional, from undergraduate to postgraduate level; the development of effective evaluation processes that actually reflect the work of this professional with a view to their ongoing education.

## Data Availability

*https://doi.org/10.48331/scielodata.SI29W0*
