# Supplementary material for: Training profile of intensive care nurses in Brazil: cross-sectional study
Source: Rev Bras Enferm. 2024 Dec 16;77(6):e20230460. doi: 10.1590/0034-7167-2023-0460 (PMC11654548; doi:10.1590/0034-7167-2023-0460)
Supplement: Translation

# Translation: Perfil formativo dos enfermeiros intensivistas no Brasil: estudo transversal

**DOI:** 10.1590/0034-7167-2023-0460pt

**Authors:** Thais Oliveira Gomes, Fernanda Berchelli Girão, Tágora do Lago Santos, Matheus Henrique Silva, Erika Azevedo Portes, Clayton Lima Melo, Marcus Vinicius Melo de Andrade

**Affiliations:** I Universidade Federal de Minas Gerais. Belo Horizonte, Minas Gerais, Brasil; II Universidade Federal de São Carlos. São Carlos, São Paulo, Brasil; III Universidade Federal do Piauí, Hospital Universitário. Teresina, Piauí, Brasil; IV Faculdade de Saúde Santa Casa BH. Belo Horizonte, Minas Gerais, Brasil; V Pontifícia Universidade Católica de Minas Gerais. Belo Horizonte, Minas Gerais, Brasil

**Keywords:** Unidades de Terapia Intensiva, Prática Profissional, Educação em Enfermagem, Educação de Pós-Graduação em Enfermagem, Enfermagem de Cuidados Críticos.

## Abstract

**Objetivos:**

descrever o perfil formativo dos enfermeiros intensivistas brasileiros.

**Métodos:**

estudo transversal realizado em duas etapas: questionário estruturado e autoaplicável; mapeamento da oferta nacional de cursos de pós-graduação *lato sensu*. Coletou-se dados sobre o perfil sociodemográfico, processo de formação e caracterização dos cursos.

**Resultados:**

na primeira etapa, obteve-se 202 respondentes. A maioria eram mulheres (79,2%), com idade entre 26 e 45 anos (80,7%), formadas há menos de 5 anos (44%), através de pós-graduação *lato sensu* (55,5%), sendo esse último aspecto marcado pela ausência de práticas em laboratórios (57,5%) e visitas guiadas (42,5%). Na segunda etapa, identificou-se 457 cursos, na modalidade de ensino presencial (58,9%), carga horária de 360 até 420 horas (51,2%), duração de até 6 meses (41,8%) e variação na subárea de formação.

**Conclusões:**

observou-se a predominância de profissionais que se formam em cursos de pós-graduação *lato sensu*, com ensino essencialmente teórico e heterogeneidade quanto à modalidade, carga horária e subárea de formação.

## INTRODUÇÃO

Doenças graves ou estados de saúde com potencial instabilidade e ameaça à vida necessitam de vigilância contínua e acompanhamento em setor específico com equipe assistencial especializada. Esses são os denominados pacientes criticamente enfermos que são internados em unidades de terapia intensiva e requerem monitorização constante e cuidados complexos, demandando uma equipe de enfermagem altamente qualificada^(1)^. As Unidades de Terapia Intensiva (UTI) são áreas destinadas à internação de pacientes e necessitam de profissionais especializados de forma contínua, bem como materiais e tecnologias específicas^(2)^.

A partir de 2020 a pandemia de COVID-19 evidenciou a escassez de enfermeiros qualificados para a atuação em terapia intensiva, além da dificuldade de se identificar profissionais com habilitação no cuidado ao paciente crítico nos cenários em que a demanda excede a oferta, como em situações de calamidade^(3)^.

A literatura demonstra que a educação permanente do enfermeiro intensivista tanto quanto sua qualificação profissional são determinantes para desfechos clínicos favoráveis dos pacientes internados em UTI, como redução no tempo de internação, no tempo para tratamento, nos custos, na mortalidade e no aumento da satisfação do paciente^(4)^. Entretanto, nem todos os enfermeiros estão aptos para atuarem no cenário da terapia intensiva e as competências e habilidades requeridas para esse tipo de assistência de enfermagem são insubstituíveis^(5)^.

No Brasil, a formação do enfermeiro intensivista se dá de três formas: (1) formação por meio de cursos de pós-graduação *lato sensu*, isto é, programas de especialização ofertados por Instituições de Ensino Superior, que possuem duração mínima de 360 horas, com a obtenção de um certificado ao fim do curso; (2) residência multiprofissional de enfermagem em terapia intensiva, conforme estabelecido pela Comissão Nacional de Residência Multiprofissional em Saúde; (3) processo de certificação profissional do enfermeiro intensivista por meio da obtenção do título de enfermeiro especialista em terapia intensiva concedido pela Associação Brasileira de Enfermagem em Terapia Intensiva (Abenti), nas modalidades adulto, pediátrica e neonatal, mediante aprovação na prova de título teórica e prática^(6)^.

O Ministério da Saúde, por meio da Resolução da Diretoria Colegiada (RDC) n 26/2012^(7)^, que altera os incisos III e IV do artigo 14 da RDC n° 07/2010^(2)^, estabelece a proporção de um enfermeiro assistencial para cada dez leitos ou fração, não exigindo um nível mínimo de qualificação para a atuação em terapia intensiva, sendo a comprovação da titulação obrigatória apenas para a coordenação de enfermagem, conforme disposto na RDC n 137/2017^(8)^.

Além disso, o Conselho Federal de Enfermagem (Cofen) regulamenta por meio da Resolução Cofen n 625/2020^(9)^ os procedimentos para registro de títulos de pós-graduação *lato sensu* e stricto sensu, bem como aquele fornecido por sociedades, associações ou colégios de especialistas em enfermagem e aprova a lista das especialidades. A referida resolução estabelece em seu artigo primeiro que o enfermeiro deverá, obrigatoriamente, promover o registro de seus títulos, não sendo esse habitualmente exigido pelos hospitais na contratação dos enfermeiros da terapia intensiva, não havendo, portanto, exigência legal da comprovação da especialidade para o exercício profissional na terapia intensiva.

Ainda com relação à formação profissional, para além do quantitativo de profissionais designadamente especialista, é preciso olhar para a qualidade e o produto dessa formação, ou seja, se o enfermeiro detentor do título de especialista em terapia intensiva é de fato capaz de prestar um cuidado complexo e de qualidade. Um estudo europeu demonstrou grande variação nos programas de especialização em pós-graduação de enfermagem em terapia intensiva no que diz respeito à duração, variações na elegibilidade, nos requisitos de avaliação e falta de acesso a recursos educacionais, o que pode impactar na qualidade do ensino e influenciar significativamente na qualidade do cuidado ofertado pelos egressos desses programas^(10)^.

Outros autores apontam que a variação no cuidado oferecido pelo enfermeiro de terapia intensiva e a falta de homogeneidade e de sistematização desse processo podem ser fatores determinantes para diferenças observadas nos resultados de pacientes internados na terapia intensiva com perfis parecidos de doenças^(11)^.

Dessa forma, considerando os diferentes tipos de formação do enfermeiro intensivista no Brasil, a não exigência da especialização para atuar como enfermeiro nesse cenário e tendo em vista que o cuidado prestado por um enfermeiro especialista em terapia intensiva tem impacto na segurança e nos desfechos clínicos favoráveis dos pacientes, emerge a necessidade de conhecer o perfil formativo do enfermeiro intensivista no Brasil, compreendendo os caminhos percorridos pelo enfermeiro intensivista ao longo da sua formação profissional, além de informações sobre quem são esses profissionais no que diz respeito a aspectos sociodemográficos, tempo e características da formação, bem como o detalhamento dos cursos de formação de enfermagem em terapia intensiva no Brasil.

## OBJETIVOS

Descrever o perfil formativo dos enfermeiros intensivistas no Brasil.

## MÉTODOS

### Aspectos éticos

O estudo foi conduzido de acordo com as diretrizes de ética nacionais e internacionais e aprovado pelo Comitê de Ética em Pesquisa da Universidade Federal de Minas Gerais, cujo parecer está anexado à presente submissão. O Consentimento Livre e Esclarecido foi obtido de todos os indivíduos envolvidos no estudo por meio online.

### Desenho e período do estudo

Trata-se de um estudo quantitativo de caráter descritivo com delineamento transversal sobre o perfil formativo dos enfermeiros intensivistas no Brasil. Os dados da pesquisa foram coletados no período de janeiro a julho de 2023. O manuscrito foi norteado a partir do referencial *STrengthening the Reporting of OBservational studies in Epidemiology* (STROBE)^(12)^.

### População, amostra e critérios de seleção

O estudo foi desenvolvido em duas etapas independentes sendo que, para a primeira, delimitou-se como público-alvo os profissionais egressos de curso de especialização de enfermagem em terapia intensiva na modalidade de residência multiprofissional em saúde ou pós-graduação *lato sensu*; aqueles que obtiveram título de enfermeiro especialista em terapia intensiva concedido por associação de especialistas registrada junto ao Conselho Federal de Enfermagem (Cofen); os enfermeiros intensivistas coordenadores de cursos de pós-graduação *lato sensu* de enfermagem em terapia intensiva e de residência multiprofissional em saúde de enfermagem em terapia intensiva. No intuito de obter um número maior de respondentes para mapear aspectos relacionados à formação, todos os enfermeiros intensivistas que preenchessem os critérios acima poderiam responder à pesquisa, desde que estivessem atuando na terapia intensiva na área de assistência, pesquisa, ensino ou gestão. Por se tratar de amostra não-probabilística, não foi previsto cálculo amostral.

Na etapa de mapeamento dos cursos de pós-graduação *lato sensu* de enfermagem em terapia intensiva cadastrados no MEC, considerou-se como critério de inclusão todos os cursos com cadastro ativo e que continham todas as informações obrigatórias do site preenchidas na íntegra.

### Protocolo do estudo

Na primeira etapa, foi realizada divulgação de questionário estruturado e autoaplicável, disponibilizado através de formulário eletrônico. Já na segunda, realizou-se mapeamento da oferta nacional de cursos de pós-graduação *lato sensu* de enfermagem em terapia intensiva cadastrados no Ministério de Educação (MEC).

Na primeira etapa, o respondente somente poderia prosseguir com o preenchimento do formulário autoaplicável caso concordasse com o Termo de Consentimento Livre e Esclarecido (TCLE). Tanto o TCLE quanto as respostas fornecidas foram disponibilizadas ao término do questionário, sendo o preenchimento limitado a uma resposta por participante. O *link* do questionário, criado no *Google Forms*, foi divulgado através de *mailing* e mídias sociais das sociedades e associações de especialistas em terapia intensiva nacional e regionais.

O questionário era constituído por 47 questões fechadas e dividido em 6 seções, a saber: seção 1: dados sociodemográficos; seção 2: pós-graduação *lato sensu* - coordenador; seção 3: residência multiprofissional em saúde - coordenador; seção 4: pós-graduação *lato sensu* - egresso; seção 5: residência multiprofissional em saúde - egresso; seção 6: titulação de enfermeiro especialista em terapia intensiva.

As questões das seções 2 a 5 abrangiam as seguintes temáticas: tempo de formação, modalidade de ensino do curso, processo seletivo para ingresso no curso, pré-requisito para ingresso no curso, carga horária, oferta de práticas em laboratório, visitas guiadas ou vivência prática em instituição hospitalar, metodologia de avaliação para obtenção do título e subárea de formação. O conteúdo das questões da seção 6 abrangia: tempo de obtenção da certificação profissional, subárea da titulação, metodologia para obtenção do título.

Considera-se pós-graduação *lato sensu* os programas de especialização com duração mínima de 360 horas, conforme Art. 44, inciso III, da Lei n 9.394, de 20 de dezembro de 1996^(13)^. Embora compreenda-se que a residência multiprofissional em saúde constitui uma modalidade de ensino de pós-graduação *lato sensu*, conforme Art. 13 da Lei n 11.129, de 30 de junho de 2005^(14)^, optou-se por descrevê-la separadamente, considerando as diferenças na carga horária, regime de dedicação e demais aspectos da formação, os quais são objeto de estudo do presente trabalho.

Para fins de padronização, entende-se titulação como o processo de certificação profissional de título de enfermeiro especialista em terapia intensiva concedido por associação de especialistas registrada junto ao Cofen.

O profissional com mais de uma formação em terapia intensiva deveria inserir essa informação no início do formulário, sendo, então, direcionado para as respostas correspondentes a cada uma das suas formações. Os coordenadores de pós-graduação *lato sensu* e residência multiprofissional, ainda que egressos de cursos, foram estimulados a informarem sobre o curso que coordenam nas seções destinadas para tal, respondendo também às questões sociodemográficas, de tempo e tipo de formação.

Na segunda etapa do estudo, foi realizada uma pesquisa no site e-MEC, na aba *Consulta Avançada*, selecionando-se a busca por *Curso de Especialização* e utilizando-se dos filtros “enfermagem em terapia intensiva” e “enfermagem em UTI”. Trata-se de dados de acesso público, provenientes da base de dados oficial dos cursos e Instituições de Ensino Superior (IES), cujas informações são inseridas pela própria IES, sendo elas responsáveis pela veracidade dos dados fornecidos. Na consulta ao e-MEC foram extraídas as seguintes informações: modalidade de ensino do curso, região de oferta, carga horária, subárea (adulto, neonatal, pediátrica ou formação conjunta com outra especialidade) e duração do curso.

### Análise dos resultados e estatística

Os dados provenientes do formulário autoaplicável e da consulta ao e-MEC foram reunidos em planilha do *Microsoft Excel* versão 16.7, sendo analisados por meio de estatística descritiva e apresentados através de tabelas em números absolutos e porcentagens.

Para a análise das seções 2 a 5 do questionário autoaplicável, as respostas dos coordenadores de pós-graduação e da residência foram analisadas conjuntamente com as dos egressos, por se tratar das mesmas questões.

## RESULTADOS

Um total de 207 participantes acessaram o questionário sendo que, desses, 202 fizeram parte da amostra final, concordando com o TCLE e respondendo ao questionário na íntegra.

Dos 202 enfermeiros intensivistas que responderam à pesquisa, 189 (93,5%) eram egressos de cursos de especialização *lato sensu*, residência multiprofissional em saúde ou profissional titulado, apenas 8 (4%) eram coordenadores de pós-graduação *lato sensu*, e 5 (2,5%) coordenadores de programas de residência multiprofissional em saúde.

A Tabela 1 apresenta a caracterização dos respondentes quanto ao perfil sociodemográfico, ao tipo e ao tempo de formação em terapia intensiva. É possível perceber que a maioria dos enfermeiros intensivistas participantes da pesquisa são mulheres (79,2%), com idade entre 26 e 45 anos (80,7%), formaram-se por meio de pós-graduação *lato sensu* (55,5%) e há menos de 5 anos (44%). No que se refere à região do país, observou-se predominância de formação nas regiões sudeste e nordeste (67,8%).

**Tabela 1:** Perfil sociodemográfico, tipo e tempo de formação em terapia intensiva dos respondentes da etapa 1 deste estudo (N=202), Belo Horizonte, Minas Gerais, Brasil, 2023

Categoria	n	(%)
Sexo Feminino Masculino	160 42	79,2 20,8
Idade Entre 18 e 25 anos Entre 26 e 35 anos Entre 36 e 45 anos Entre 46 e 55 anos Acima de 55 anos	10 64 99 25 4	5 31,7 49 12,3 2
Região do país em que formou Sul Sudeste Norte Nordeste Centro oeste	23 73 24 64 18	11,4 36,1 11,9 31,7 8,9
Tipo de formação em terapia intensiva Pós-graduação *lato sensu* Residência multiprofissional em saúde Titulação Mais de uma formação^*^	112 30 38 22	55,5 14,8 18,8 10,9
Tempo de formação em terapia intensiva Há menos de 5 anos Entre 5 e 10 anos Acima de 11 anos	100 67 60	44 29,5 26,5

* *Mais de uma formação: pós-graduação lato sensu e titulação ou pós-graduação lato sensu e residência multiprofissional ou residência multiprofissional e titulação ou as três modalidades.*

A seguir, são apresentados os detalhamentos relacionados à cada um dos tipos de formação em terapia intensiva (pós-graduação *lato sensu*, residência multiprofissional em saúde e titulação) relatadas pelos respondentes na etapa 1 deste trabalho. Considerando a possibilidade de informar mais de uma formação em terapia intensiva, foram obtidas 227 respostas do total de 202 respondentes, sendo 127 referentes à pós-graduação *lato sensu*, 40 à residência multiprofissional e 60 à titulação.

Com relação à formação por meio da pós-graduação *lato sensu*, a Tabela 2 evidencia que os cursos foram marcados pelo ensino presencial (78,7%), com carga horária variando de 360 até 420 horas (76,4%) e ausência de oferta de práticas em laboratórios (57,5%) e visitas guiadas (42,5%), apresentando um caráter essencialmente teórico, o qual também é percebido através da metodologia de avaliação para obtenção do título. Nesse caso, permitiu-se ao respondente a marcação de mais de um item, tendo sido citadas a apresentação de Trabalho de Conclusão de Curso (62%), a submissão de artigo científico (11%), frequência mínima nas aulas (22,5%) e outras como provas, aprovação em todas as disciplinas ofertadas e nota média (4,5%). Embora tenha sido constatada uma predominância de cursos destinados à formação específica em uma subárea - adulto, neonatal ou pediátrica - a qual esteve presente em 70,1% das respostas, é perceptível a presença de cursos com formação em mais de uma subárea, tendo sido citada a formação conjunta em terapia intensiva adulto, neonatal e pediátrica (13,4%) e a formação associada a áreas afins à terapia intensiva, como cardiologia, urgência e emergência e trauma (16,5%).

**Tabela 2:** Caracterização dos cursos de pós-graduação *lato sensu* de enfermagem em terapia intensiva identificados na etapa 1 do estudo (n=127), Belo Horizonte, Minas Gerais, Brasil, 2023

Descrição	n	(%)
Modalidade de ensino Presencial À distância Híbrido	100 14 13	78,7 11 10,3
Processo seletivo para ingresso no curso Sim Não	83 44	65,4 34,6
Carga horária do curso Até 360 horas Entre 360 e 420 horas Acima de 420 horas	40 57 30	31,6 44,8 23,6
Práticas em laboratório Sim Não	54 73	42,5 57,5
Vivência prática ou visita guiada Sim (optativa) Sim (obrigatória) Sim (visitas em disciplinas específicas) Não	5 34 34 54	4,1 26,7 26,7 42,5
Formação específica em uma subárea Sim Não (adulto, neonatal e pediátrica) Não (terapia intensiva e área afim)	89 17 21	70,1 13,4 16,5
Metodologia da avaliação para obtenção do título Apresentação de trabalho de conclusão de curso Submissão de artigo científico em periódico indexado Frequência mínima nas aulas Outras^*^	108 19 39 8	62 11 22,5 4,5

* *Outras: Provas, aprovação em todas as disciplinas ofertadas, nota média.*

Com relação à formação por meio de residência multiprofissional em saúde, das 40 respostas obtidas, 100% dos enfermeiros intensivistas participaram de processo seletivo para ingresso no curso, tendo sido relatada a análise de currículo, entrevista e prova de múltipla escolha e/ou dissertativa. Vinte e cinco (62,5%) dos enfermeiros relataram não haver oferta de práticas em laboratório. Quanto à subárea de especialidade, identificou-se uma formação predominante na subárea de terapia intensiva adulto: 25 (62,5%), seguida de formação mista em terapia intensiva adulto, neonatal e pediátrica (14%), específica em neonatal e pediátrica (9%), bem como em terapia intensiva e áreas afins (14%), nos mesmos moldes do que foi identificado na pós-graduação *lato sensu*.

Já na formação por meio da titulação, das 60 respostas obtidas, identificou-se uma predominância de formação na subárea adulto 50 (83,3%), seguida da pediátrica 6 (10%) e neonatal 4 (6,7%).

A seguir são apresentados os dados referentes à etapa 2 deste trabalho. Foram identificados 457 cursos de pós-graduação *lato sensu* de enfermagem em terapia intensiva com situação ativa no e-MEC. A modalidade de ensino majoritária foi a presencial (58,9%), com uma predominância de cursos na região sudeste (48,4%), com carga horária de 360 até 420 horas (51,2%) e duração preponderante de até 6 meses (41,8%). Quando informada, notou-se uma grande variação na descrição da subárea do curso, tendo sido identificados cursos destinados exclusivamente à formação em adulto (9,8%), em neonatologia (3,3%), bem como em neonatologia e pediatria concomitantemente (20,8%), também em adulto, neonatologia e pediatria (3,1%) e associados a outras áreas de cuidado ao paciente crítico, como urgência e emergência, trauma, cardiologia e neurologia (14,2%).

Os resultados da segunda etapa do estudo são apresentados na Tabela 3.

**Tabela 3:** Dados dos cursos de pós-graduação *lato sensu* de enfermagem em terapia intensiva com situação ativa no e-MEC identificados na etapa 2 do estudo (N=457), Belo Horizonte, Minas Gerais, Brasil, 2023

Descrição dos cursos	n	(%)
Modalidade de ensino Presencial Distância	269 188	58,9 41,1
Região de oferta Sul Sudeste Norte Nordeste Centro oeste Mais de uma região	65 221 31 63 33 44	14,2 48,4 6,8 13,8 7,2 9,6
Carga horária Até 360 horas Entre 360 e 420 horas Acima de 420 horas Acima de 5000 horas	130 104 221 2	28,4 22,8 48,4 0,4
Duração Até 6 meses Entre 6 e 12 meses Entre 13 e 18 meses Entre 19 e 24 meses	191 105 117 43	41,8 23 25,6 9,4
Subárea do curso Não informada Adulto Neonatal Pediátrico Neonatal e pediátrico Adulto, neonatal e pediátrico Adulto e pediátrico Adulto e neonatal Terapia intensiva e outras subespecialidades (urgência e emergência, trauma, cardiologia, neurologia)	216 45 15 0 95 14 1 6 65	47,3 9,8 3,3 0 20,8 3,1 0,2 1,3 14,2

## DISCUSSÃO

Em relação ao perfil sociodemográfico, os dados deste estudo revelaram que a maioria dos participantes eram mulheres com idade entre 36 e 45 anos. Esses resultados são semelhantes aos relatados por estudo multicêntrico sobre o perfil dos profissionais de enfermagem em terapia intensiva realizado na América do Sul, com participação de Argentina, Colômbia, Peru e Brasil, onde a prevalência feminina nos quatro países foi de 83% e no Brasil foi 79%, enquanto a idade mediana global foi 36 anos e brasileira 29 anos^(15)^. Em países mais distantes como o Japão, a predominância de composição feminina (77,7%) e jovem com faixa etária de 20 a 29 anos também é encontrada, embora existam aspectos econômicos e culturais diferentes^(16)^.

Os dados deste estudo demonstram que os enfermeiros intensivistas brasileiros são, em sua maioria, jovens na especialidade, possuindo tempo de formação em terapia intensiva de até 5 anos. O tempo de formação em terapia intensiva é fundamental para que o enfermeiro intensivista desenvolva habilidades de raciocínio e julgamento clínico, fortalecendo sua prática e promovendo maior segurança para sua atuação profissional^(15)^. Sobre o tempo de atuação em terapia intensiva e a manutenção de enfermeiros atuando na especialidade, um estudo americano discute a dificuldade em reter enfermeiros na terapia intensiva, o que pode estar associado a fatores como burnout e sobrecarga de trabalho, circunstâncias agravadas pela pressão imposta durante a pandemia de COVID-19, nessas unidades^(5)^. Alguns autores problematizam que há uma tendência do enfermeiro intensivista fazer transição para cargos fora das unidades de terapia intensiva na medida em que avançam na sua formação^(17)^. Assim, são sugeridas estratégias para a retenção de enfermeiros intensivistas trabalhando na especialidade, nos diferentes âmbitos de atuação (gestão, ensino/pesquisa e/ou assistência), tais quais: reconhecimento, respeito e valorização do papel importante e dos níveis elevados de treinamento necessários para a atuação nesse cenário; participação ativa nas atividades da unidade, com envolvimento em tomadas de decisões complexas como em situações de fim de vida, manejo da dor e conforto, protocolos guiados, *rounds* multidisciplinares; estímulo ao desenvolvimento intelectual e profissional; encorajamento de oportunidades de aprendizagem e envolvimento em atividades de formação como residência; além de formação contínua em liderança com instruções sobre *feedback*, procurando promover crescimento mútuo na equipe, dentre outras^(5)^.

Nessa seara, é importante citar que, embora em um quantitativo menor, identificou-se um número razoável de enfermeiros com mais de um tipo de formação em terapia intensiva (pós-graduação *lato sensu* e titulação ou pós-graduação *lato sensu* e residência multiprofissional ou, ainda, residência multiprofissional e titulação ou as três modalidades). Esse achado pode estar relacionado ao fato de ter sido permitida a manifestação de enfermeiros vinculados ao ensino, pesquisa e gestão da terapia intensiva, além daqueles inseridos em atividades assistenciais. Estudo transversal sobre a avaliação das competências do enfermeiro em atuação na terapia intensiva de hospitais terciários no Japão identificou que os profissionais com um maior número de títulos de terapia intensiva obtiveram melhores resultados nas competências relacionadas à tomada de decisão, colaboração e intervenções de enfermagem^(16)^. Portanto, a manutenção desse profissional em atividades relacionadas ao cuidado ao paciente crítico, seja na assistência a beira leito, seja em cargos de gestão ou em posições de ensino e pesquisa voltadas para a área é fundamental para garantir que o cuidado complexo e o conhecimento avançado necessário para atuação na terapia intensiva sejam garantidos^(17)^.

No que concerne o perfil formativo, considerando os três tipos de formação para enfermagem em terapia intensiva no Brasil, há predominância da formação por meio de pós-graduação *lato sensu*. Percebe-se também que a maior parte dos cursos não possui carga horária de práticas em laboratório, evidenciando uma formação essencialmente teórica, o que também pode ser verificado na metodologia de avaliação para obtenção do título. A vivência prática e/ou visitas guiadas em serviços de saúde se aplica principalmente ao modelo de Residência multiprofissional em saúde, pela própria natureza que a caracteriza como uma formação em serviço. Porém, neste estudo, esse tipo de formação foi substancialmente menor se comparada às demais.

Ainda no que diz respeito ao perfil formativo, apesar da modalidade de ensino presencial ter sido identificada nas duas etapas do trabalho como a oferta de ensino de pós-graduação *lato sensu* majoritária, chama atenção o percentual elevado de oferta de cursos à distância identificados na etapa 2, os quais apresentam uma curta duração, geralmente de até 6 meses, com carga horária variando de 360 até 420 horas.

O número expressivo de cursos de pós-graduação *lato sensu* na modalidade à distância, somada à carência de cursos com vivência prática ou visitas guiadas são fatores preocupantes no que se refere à qualidade do ensino e no desenvolvimento de competências do enfermeiro intensivista. Adquirir experiência é parte importante no desenvolvimento da intuição e da competência profissional, pois permite ao enfermeiro antecipar a evolução de um quadro clínico e basear as decisões em experiências anteriores com situações semelhantes^(18)^. A segurança no gerenciamento de situações depende da experiência do enfermeiro. Formadores e educadores em cuidados intensivos precisam considerar a concepção de programas práticos para enfermeiros inexperientes, que incluam a exposição intencional a várias situações clínicas com risco de vida inerentes à enfermagem em terapia intensiva^(18)^. Para além disso, não é possível, em formações à distância e sem prática em serviço, aprimorar competências como o trabalho em equipe, o manejo de influências demográficas, culturais, relações interpessoais com pacientes de UTI e seus familiares, ou gerenciamento de tecnologias^(18)^. Assim, há que se considerar que, sendo a pós-graduação *lato sensu* o tipo de formação predominante e majoritariamente sem oferta de atividade prática, existe a possibilidade de insuficiência dessa modalidade de formação no que concerne à aquisição de competências para atuação em uma unidade de terapia intensiva.

Nos resultados aqui encontrados, é notório que existe uma heterogeneidade na formação do enfermeiro em terapia intensiva no Brasil, desde a forma de acesso à oportunidade de especialização, que pode selecionar profissionais para formação por meio de provas, até cursos que somente requerem graduação prévia como requisito, passando por variações na duração, carga horária, modalidade de ensino e subárea de formação. De forma similar, um estudo europeu identificou a falta de um padrão nacional de ensino e treinamento como um problema na maioria dos países^(19)^. As dificuldades vão desde requisitos de elegibilidade e duração do curso, que varia de 240 horas a 24 meses, sem consistência em como os alunos foram avaliados e qualificados para a concessão da formação, seguidas da falta de proteção para o título, horário de trabalho do aluno limitador que impacta o ensino e treinamento na UTI, e a falta de acesso à recursos em educação^(19)^. Ainda com relação ao acesso à especialização, um estudo australiano identificou que a maior parte dos cursos não exigia como pré-requisito ter experiência clínica anterior para ingresso na formação, o que se assemelha à realidade brasileira encontrada neste estudo, tendo como exceção somente a prova de título^(20)^.

No que se refere à formação em mais de uma subárea, um achado importante deste estudo foi a heterogeneidade da subárea de formação em terapia intensiva, passando por cursos com formação conjunta em adulto, neonatologia e pediatria, até cursos de especialização em terapia intensiva e áreas afins, como cardiologia, neurologia, urgência, emergência e trauma. Autores australianos revelaram que, no país, existem várias interpretações do que compreende uma qualificação de cuidados intensivos, havendo pós-graduações em que o conteúdo era lançado de forma ampla para atender às áreas de terapia intensiva, cuidados cardíacos ou, para alguns, uma combinação de enfermagem de emergência, enfermagem de alta dependência e/ou enfermagem do trauma^(20)^.

Nesse aspecto, cabe problematizar a generalização de uma especialização, afinal o seu objetivo deveria ser afunilar o conhecimento e adquirir competências específicas para um determinado contexto de atuação. Contudo, o que se percebe é um movimento de contemplar múltiplas clínicas num único escopo de formação, o que aponta para o risco de, ao tentar abarcar muitas disciplinas, a formação ofertada seja cada vez mais generalista e menos capaz de promover, de fato, uma especialização. Ainda nessa seara, outro achado que merece reflexão é a carga horária inferior a 420 horas na maior parte dos cursos de pós-graduação *lato sensu*. Assim, embora o MEC defina uma carga horária mínima de 360 horas, problematiza-se sobre qual seria a carga horária suficiente para a abrangência de mais de uma área de conhecimento e sua especificidade.

O Conselho Federal de Enfermagem, no artigo 1^0^ da Resolução Nº 581/2018^(21)^ e seus desdobramentos (COFEN Nº 625/2020; COFEN NºS 065/2021 e120/2021), aponta a obrigatoriedade de registro dos títulos de pós-graduação *lato sensu* e stricto sensu (este último na modalidade profissionalizante), no Conselho Regional de sua jurisdição. Entretanto, nem tal registro no Conselho, nem a titulação de especialista são exigidas pelas instituições de saúde para empregar o enfermeiro que atuará nas unidades de terapia intensiva, com exceção do coordenador de enfermagem.

O anexo das legislações mencionadas acima, ao definir as áreas e subáreas de conhecimento para registro dos títulos junto ao Conselho Regional de Enfermagem, limita o registro do título às áreas elencadas no instrumento. Assim, embora existam cursos que sejam multiáreas, conforme identificados na pesquisa, o enfermeiro só poderá registrar nas áreas e subáreas elencadas no anexo da Resolução Nº 581/2018^(21)^. As subáreas permitidas no registro da titulação de Terapia Intensiva, item 43 do anexo da referida Resolução são somente: a) Adulto, b) Cardiológica, c) Neurológica, d) Pediátrica, e) Neonatologia, não podendo-se acumular subáreas com um único curso de especialização.

Um achado deste estudo que merece atenção é a predominância de formação nas regiões sudeste e nordeste identificadas na etapa 1, bem como o número significativo de cursos ofertados nessas localidades verificado na etapa 2. Tal fato pode estar associado à disponibilidade de leitos de terapia intensiva nas referidas regiões, o que pode levar a uma maior procura de formação específica para esse tipo de cuidado. Os dados do Censo da Associação de Medicina Intensiva Brasileira (AMIB) sobre a distribuição de leitos de terapia intensiva no Brasil em Janeiro de 2023 revelaram um total de 22.618 leitos na região sudeste e 9.429 na região nordeste, sendo o primeiro e o segundo lugar, respectivamente, em número absoluto de distribuição de leitos de terapia intensiva públicos e privados no país^(22)^.

### Limitações do estudo

Este estudo apresenta como limitação o número pequeno de respondentes da etapa 1, na qual foram obtidos dados referentes à formação de 202 enfermeiros intensivistas. Entretanto, segundo dados oficiais obtidos através de contato com os canais de comunicação formal da Abenti em junho/2023, o número total de enfermeiros titulados por meio do processo de certificação e obtenção do título de enfermeiro especialista em terapia intensiva nas modalidades adulto, pediátrica e neonatal é de 405 profissionais. Dados do Cofen sobre o quantitativo de enfermeiros com cadastro do título de especialista em terapia intensiva nas modalidades adulto, neonatal e pediátrico em 31 de maio de 2023 era de 11.605 profissionais, segundo informações obtidas por meio de solicitação registrada no sistema de Ouvidoria do Cofen. Porém, trata-se do quantitativo global de enfermeiros com título cadastrado sem filtro temporal e que pode, portanto, englobar profissionais que não se encontram em situação ativa, como profissionais aposentados.

### Contribuições para a área da Enfermagem

Trata-se, ao nosso conhecimento, do primeiro estudo que apresenta dados sobre o quantitativo de enfermeiros intensivistas, bem como o perfil formativo desse especialista no Brasil.

Outro aspecto relevante deste trabalho é que os dados sobre a formação por meio da pós-graduação *lato sensu*, identificados na etapa 1, concernentes à carga horária, modalidade de ensino, região de oferta e subárea, são similares àqueles encontrados na etapa 2, quando foram mapeados todos os cursos de pós-graduação *lato sensu* com cadastro ativo no e-MEC. Assim, pode-se dizer que há solidez e coerência nos dados obtidos na condução das duas etapas do trabalho, sendo as informações da etapa 1 reafirmadas na etapa 2, o que faz sentido, pois sendo a pós-graduação *lato sensu* o tipo de formação predominante, a caracterização desta por meio dos dados oficiais do MEC clarificou os achados da etapa 1.

Assim, o presente trabalho não somente abre caminhos para novos estudos, mas levanta outras questões adjacentes ao tema e que expliquem, entre outras questões, como a formação desenha a competência técnica que de fato se requer para a atuação do enfermeiro na terapia intensiva.

## CONCLUSÕES

Este estudo descreveu o perfil formativo do enfermeiro intensivista no Brasil no que diz respeito ao tipo de formação predominante no país, além de aspectos sociodemográficos e do tempo de formação desse profissional. Também foram apresentados detalhamentos relacionados a cada um dos tipos de formação em terapia intensiva quanto à carga horária, duração, modalidade de ensino, ingresso no curso, metodologia de avaliação e subárea de formação.

A predominância de profissionais que formam em cursos de pós-graduação *lato sensu* encontrados neste estudo, com oferta de ensino essencialmente teórico, bem como a sua heterogeneidade no que diz respeito à modalidade de ensino, duração, carga horária e subárea de formação demonstram a necessidade de se definir as competências profissionais do enfermeiro intensivista no Brasil.

Dessa maneira, torna-se imperativo promover avanços para uma formação profissional baseada em competências, que oportunize um processo contínuo de avaliação do enfermeiro intensivista com base nos conhecimentos, habilidades, atitudes e valores esperados para sua atuação na prática clínica. Ademais, entende-se a necessidade de se descrever as competências profissionais do enfermeiro intensivista, com vistas a contribuir para: uma melhor definição do papel e das atribuições no dia-a-dia da sua atuação; definição dos requisitos mínimos essenciais para a sua atuação profissional na terapia intensiva; a padronização de uma linguagem comum para a sua formação; a compreensão das lacunas existentes na formação profissional, desde a graduação até a pós-graduação; o desenvolvimento de processos avaliativos efetivos que de fato reflitam a atuação profissional com vistas à sua educação permanente.

## DISPONIBILIDADE DE DADOS E MATERIAL

*https://doi.org/10.48331/scielodata.SI29W0*
